# Promoting the Mental Health of Hospital Professionals Through a Workplace-Based Mindfulness Intervention: Protocol for a Randomized Controlled Pilot Trial With a Realist Evaluation (PROMIND)

**DOI:** 10.2196/80725

**Published:** 2026-08-18

**Authors:** Ludivine Nohales, Sandrine Touzet, Anne Termoz, Laurent Remontet, Lou-Anne Audouard, Marion Trousselard, Emmanuel Fort, Nathalie Streichenberger, Pascal Delamillieure, Antoine Lutz, Delphine Maucort-Boulch, Jean-Baptiste Fassier, PROMIND Study Group

**Affiliations:** 1 Occupational Health and Medicine Department- CRPPE, Hospices Civils de Lyon, place arsonval, LYON, Rhône-Alpes, 69003, France, 33 472116340; 2 UMRESTTE UMR T 9405, Université Claude Bernard Lyon 1, LYON, Auvergne-Rhône-Alpes, France; 3 RESHAPE, Université Claude Bernard Lyon 1, LYON, France; 4 Service Recherche et Epidémiologie Clinique, Pole de Santé Publique, Hospices Civils de Lyon, LYON, France; 5 Service Biostatistiques-Bioinformatiques, Pole de Santé Publique, Hospices Civils de Lyon, LYON, France; 6 UMR 5558, LBBE - Laboratoire de Biométrie et Biologie Evolutive, Université Claude Bernard Lyon 1, Villeurbanne, France; 7 Occupational Health and Medicine Department, Hospices Civils de Lyon, Lyon, France; 8 Institut de recherche Biomédicale des Armées, BRETIGNY SUR ORGE, France; 9 UMR 1319, INSPIIRE, Centre Hospitalier Régional et Universitaire de Nancy, NANCY, France; 10 Service d'anatomopathologie, CBPE, Hospices Civils de Lyon, LYON, France; 11 CNRS UMR 5261, INSERM U1315, Université Claude Bernard Lyon 1, LYON, France; 12 Service de psychiatrie adulte, Centre Hospitalo-Universitaire de Caen, CAEN, France; 13 UMR 6301 ISTCT, Université de Caen Basse-Normandie, CAEN, France; 14 INSERM U1028, CNRS UMR5292, Eduwell, Lyon 1 University, LYON, France; 15 see Acknowledgments

**Keywords:** community-based participatory research, mindfulness, feasibility studies, mental health, flourishing at work, health workforce

## Abstract

**Background:**

Hospital professionals face mental health challenges caused by the high demands of their work. Mindfulness meditation (MM) is a nonpharmacological practice with promising effects in promoting mental health and well-being across various hospital professionals. However, the benefits obtained from its implementation in the context of real work in hospital departments are understudied.

**Objective:**

The aims of this pilot study are to implement and evaluate, at both the individual and collective levels, the exploratory outcomes of a workplace-based mindfulness intervention developed participatively for hospital teams in a French university hospital. The feasibility of the implementation of the intervention, the influence of the work environment, and the mechanisms through which it produces its effects will also be assessed.

**Methods:**

For this prospective interventional study, a preliminary co-construction with the stakeholders (a task force with professionals and management) made it possible to develop and adapt the intervention to the needs and capacities of the professionals, using the intervention mapping model for health program planning. Inclusion and an MM information meeting were proposed to 8 hospital departments, which were then randomized into 2 parallel cluster groups (intervention or control group). In the intervention group, an MM test session was proposed before committing to the MM program (10 one-hour sessions) for a maximum of 15 professionals per department. A convergent mixed methods design with concomitant quantitative and qualitative data collection was used, before integration of findings. Quantitative evaluation was based on a comparison of “before-after” measurements, with a comparison between intervention and control groups. The primary exploratory outcome was the evolution of psychological flourishing at work, which was measured using the adapted and validated French-language scale Psychological Flourishing at Work (EEPMT-8), to which the Professional Life Satisfaction Scale (ÉSVP-5) was added, to form a new 13-item Psychological Flourishing at Work Scale (EEPMT-13). Realist evaluation by qualitative methods explored the influence of contexts on the implementation (barrier and facilitators), and the links between context, mechanisms, and effects of MM. The study was approved by the Sud-Est VI ethics committee (Clermont Ferrand, France).

**Results:**

Funding was obtained in October 2021 and June 2024. This study was conducted from May 2024 to June 2025. As of May 2024, 108 participants were enrolled. Analyses are in progress. Results are expected to be published by the end of 2026.

**Conclusions:**

Participative approaches are essential for a challenged, tailored implementation of a workplace-based mindfulness intervention in hospital teams. The evaluation of the intervention with mixed methods and the characterization of both individual and organizational levels are aligned with the recommendations to evaluate a complex intervention in a workplace; challenges include the use of an innovative primary endpoint and methodological pluralism to plan for scalability at a subsequent stage.

## Introduction

### Mental Health of Hospital Professionals

Mental health is a state of well-being in which a person can realize their potential, cope with the stresses of life, work productively, and contribute to the life of their community. It refers to a continuum that spans the promotion of well-being, the prevention of mental disorders, and the treatment and rehabilitation of people with these disorders (World Health Organization) [1]. The prevention of psychosocial risks coupled with the promotion of mental health in the workplace is a major challenge [1,2]. Hospital professionals face major mental health challenges due to the demands of their profession, characterized by a heavy workload and frequent confrontation with human distress [3-7]. The frequency of mental health difficulties among hospital professionals is significant at all stages (malaise, distress, and mental illnesses). A meta-analysis found that approximately 30% of caregivers have anxiety, 30% have depression, 30% experience psychotraumatic symptoms, and 45% have sleep disorders [8]. At the other end of the mental health spectrum, the concept of psychological flourishing is conceptualized as a positive mental health state, encompassing optimal psychological functioning in relation to various life domains [9-12]. The contribution of teamwork to mental health has been demonstrated; in this context, teamwork is defined as a collective construction enabling a shared vision of professional standards of each professional concerning their own health and safety and the management of their activity [13]. Strong team cohesion and social support networks may serve as protective factors against psychological strain [14].

### Mindfulness Meditation

The contribution to mental health and well-being of complementary health practices is gaining evidence and interest among users, professionals, and academics. The integration of conventional and complementary health practices is of particular interest [15]. Nonpharmacological interventions have evidence-based impacts [16] that are interesting for promoting mental health. The effects of mindfulness meditation (MM) in health have been extensively studied. Mindfulness is defined as a state of awareness that arises when one intentionally and nonjudgmentally pays attention to the experience of the present moment [17]. It can be trained through formal and informal mindfulness practices. Typical programs of mindfulness-based stress reduction (MBSR) or mindfulness-based cognitive therapy (MBCT) include 8 weekly in-person sessions lasting 2 to 2.5 hours, a half-day or full-day session, and a recommended 45-minute daily individual practice [18-20]. Available in a variety of protocols, it is reported to improve resilience [21] and physical and mental well-being (stress, anxiety, burnout, and affects) [22-25]. In workplace contexts, the beneficial effects of a mindfulness-based intervention (MBI) include better stress adaptation [26], fewer interpersonal conflicts, as well as improved care and patient relationships (eg, medical errors) [27-31]. More broadly, impacts are observed on relational behaviors (antisocial and prosocial) [32,33], teamwork [30,34], and performance and job satisfaction [35,36]. Managers may benefit from a strengthened aspiration to lead, with a vision more fully committed to serving others and their colleagues [37,38]. Thus, MM could be a promising practice to promote mental well-being and flourishing at work for hospital professionals [39]. However, although studies have identified benefits of an MBI at a collective level in workplace contexts, no clear explanation for such effects has been found [34,38]. Therefore, it has been recommended to explore in more detail the mechanisms by which the effects of MM at a collective level can be activated depending on the context [40].

### Implementation Challenges

The time constraints of typical MBIs, combined with those related to professionals’ schedules and training costs, can limit the participation of individuals, teams, and organizations in these programs [41]. Implementing such an MBI in a hospital setting is a particularly challenging endeavor, especially in the context of health care professional shortages and absenteeism. In addition, support from hospital leadership is essential for successful implementation [42]. The UK Medical Research Council (MRC) recommends that complex interventions be developed using participatory approaches [43,44]; the influence of the work environment on the implementation of MBIs in the workplace has been reported, with identification of factors that can either facilitate or hinder both implementation and effectiveness [42,45]. The benefits of mindfulness in the workplace thus depend on the intervention being implemented in a supportive context; it is therefore recommended that proposed interventions be closely aligned with the organization’s values [42]. Furthermore, ensuring participants’ sense of psychological safety during an intervention at work is essential. Psychological safety is a key mechanism potentially activated by MBIs in workplace contexts. This in turn facilitates interpersonal trust and mutual respect within the team or, conversely, feelings of relational insecurity [38,46].

### Evaluation Challenges

MBIs in the workplace should be considered as complex interventions, given the different levels at which effects may occur (both individual and collective), as well as the variety of potential outcomes that may be observed. Methodological pluralism is a strong recommendation of the MRC for the evaluation of complex interventions, to evaluate from multiple perspectives and to better capture the mechanisms through which effects are produced [44]. Two major complementary paradigms are thus mobilized: the intervention epidemiology paradigm and the realist evaluation paradigm [43,47-49]. The latter [42] enables the assessment of the variation of effects and underlying mechanisms according to each context (hospital department) through the identification of context-mechanism-outcome configurations (CMOCs). This evaluation aims to understand how, why, for whom, and under what circumstances a given intervention is expected to produce specific results, by examining underlying mechanisms, processes, and cause-and-effect relationships. This recommended approach allows to refine the understanding of how the effects of a complex intervention—which also mobilizes social interactions—vary according to the context in which it is implemented [44]. In the area of the MBIs, there is little high-quality assessment of potential benefits and risks. Because of the difficulties of participation mentioned above, short interventions are studied, with varied content (duration and modalities), producing variable results [41]. The current knowledge on the barriers and facilitators to the implementation of MBIs in health care settings and for health care professionals justifies ensuring the implementation feasibility in such a context. The benefits obtained from its implementation in the context of real work in hospital departments are understudied [50]. No published study has examined the feasibility challenges related to the implementation of an MBI in hospital departments within the French context.

### Context of the PROMIND Study

The Hospices Civils of Lyon (HCL; France) is a consortium of university-affiliated hospital facilities in France, comprising 13 different institutions and employing approximately 24,000 people [51]. In France, in accordance with the French Labor Code, employers are legally responsible for assessing occupational risks and protecting the physical and mental health of their employees. Given the presence of multiple psychosocial risk factors that may affect employees’ mental health, the HCL, together with the Occupational Health Department, initiated and developed the PROMIND study.

### Objectives

**Figure 1.**
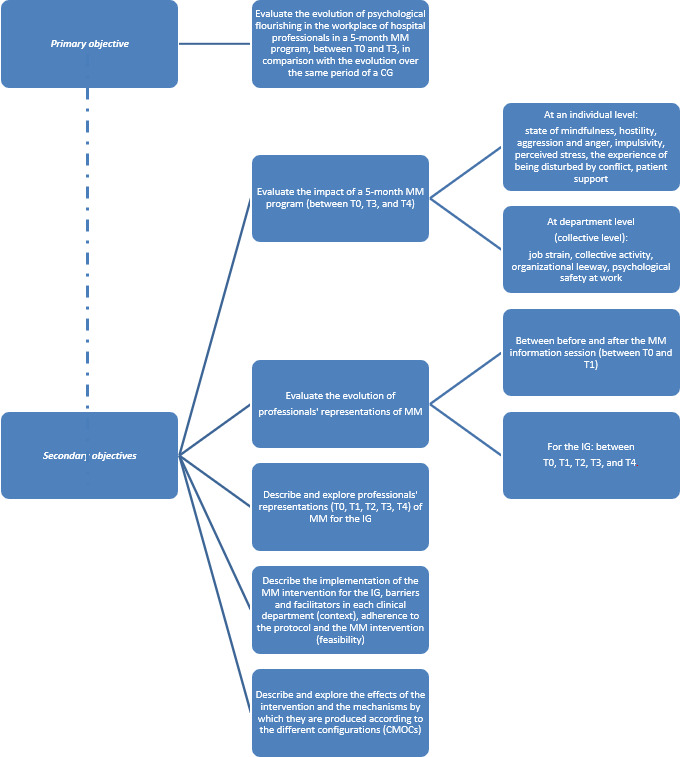
Objectives of the PROMIND study. CG: control group; CMOC: context-mechanism-outcome configuration; IG: intervention group; MM: mindfulness meditation; T0: the baseline; T1: after the MM information meeting/before the MM test session; T2: after the MM test session; T3: after the end of the MM program; T4: 3 months after the end of the MM program.

The aims of this pilot study are to implement a workplace-based mindfulness intervention and evaluate exploratory individual and collective outcomes among hospital professionals. The feasibility of the implementation of the intervention, the influence of the work environment, and the mechanisms through which it produces its effects are also assessed (Figure 1).

## Methods

### Trial Design

**Figure 2.**
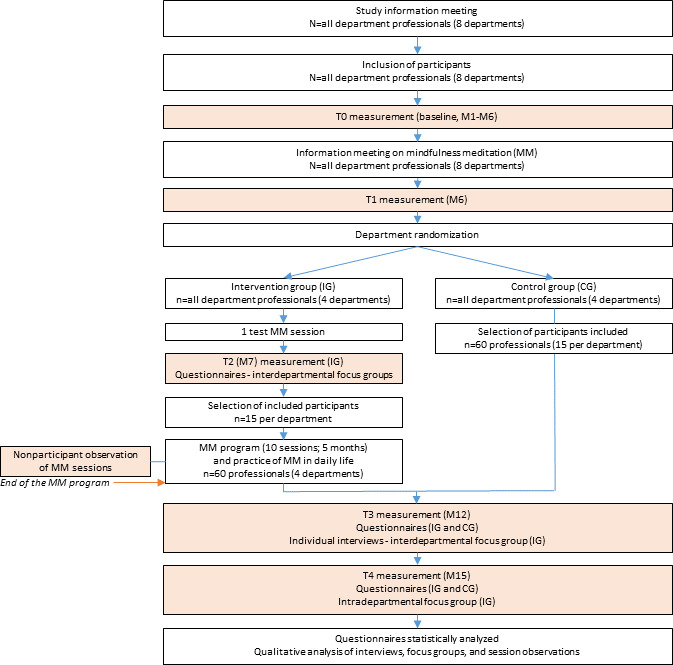
PROMIND flowchart. M: months; MM: mindfulness meditation; T0: the baseline; T1: after the MM information meeting/before the MM test session; T2: after the MM test session; T3: after the end of the MM program; T4: 3 months after the end of the MM program.

This prospective interventional pilot study, with 2 parallel groups randomized in clusters and open-label design, is being conducted at the university hospitals of Lyon (HCL), France (Figure 2). The protocol presented herein covers the step of the intervention mapping model aimed to implement (step 5) and evaluate (step 6) the intervention.

### Participatory Intervention Development

As recommended for developing complex interventions [43,44], a participatory approach was used before the study to develop and tailor the intervention to the needs and capabilities of the users, using the intervention mapping protocol (steps 1‐4) [52-54]. This stage was approved by the ethics committee of the university of Lyon (*Comité d’éthique de la recherche de l’Université de Lyon*; N/ref: 2022-04-14-005). Eight volunteer departments were identified during the intervention development step and matched in pairs by specialty and by geographical location among the hospitals of the HCL for control purposes (ie, medicine, geriatrics, anesthesia-intensive care, and intensive care/neonatology). A task force (34 people) included a committee of participants’ representatives (COPR) with “ambassadors” from participating departments (nurse assistants; nurses; physicians; department managers such as the department head physician, the head nurse, and the administrative manager; administrative professionals; workers’ representatives; and hospital managers), mindfulness experts, and PROMIND researchers. The COPR (24 people) attended a project presentation meeting followed by individual interviews and 4 participative workshops, including guided MM practice, delivered by an expert (a hospital physician, a university professor, and an experienced MM trainer). Qualitative data were collected, the analysis of which allowed to understand the needs and representations of professionals, including perceived barriers and facilitators at the individual, team, and organization levels. After the needs assessment and a literature review, the modalities of the intervention (frequency, location, and content) were decided in close collaboration within the task force. The executive management of the HCL was mobilized as a key stakeholder from the start of the development of the intervention, to support the participation of department managers and teams, including communication, funding, and material assistance: premises were made available, and the study was considered as work and training time, as were trips made specifically for the study. The objective of the co-construction step was to adapt the intervention to the context, improve the adherence to the project, and improve the understanding of MM practice.

### Initial Recruitment

The study was proposed to all professionals in the 8 departments, on a voluntary basis. The call for participation and the collection of consent were carried out after the study information meeting. Participants were not aware of their department’s allocation group at the time of enrollment.

The inclusion criteria for the study were being employed by the hospital and working in one of the participating departments, being aged ≥18 years, and being willing to give written informed consent. The exclusion criteria were having a self-reported neuropsychiatric disorder with current severe clinical instability (severe depressive or affective disorder, severe anxiety disorder, psychotraumatic disorder, severe dissociative disorder, psychotic disorder, epilepsy, etc), being unable to understand or write in French, and being pregnant or breastfeeding.

Premature study exit criteria were acute neuropsychiatric symptoms uncontrolled during the study (eg, severe dissociation, major anxiety, and severe behavioral disturbance) and withdrawal of consent to participate. Participants who left the study were not replaced. Simultaneous participation in another study was not prohibited unless it involved the use of a psychotropic or psychotropic-like drug or proposed psychiatric or psychological follow-up.

### The MM Information Meeting

The MM information meeting was opened to the professionals in the 8 participating departments. The definition of MM and various representations of this concept were discussed. Media were used, including testimonies from experts, COPR professionals, and peers and a participatory game.

### Allocation of the Groups

Randomization was carried out by the biostatistics team after the MM information meeting. Randomization was carried out at the department level and stratified by department type (medicine, geriatrics, anesthesia-intensive care, and intensive care/neonatology) to ensure that each department type was represented in both the intervention group (4 departments) and control group (4 departments). Cluster randomization was chosen to prevent contamination within departments, as professionals work closely together.

### The MM Practice Intervention

- MM test session: after randomization of the departments, an MM test session was proposed to all professionals of each department allocated in the intervention group.
- Practice program: the MM program consisted of 10 one-hour sessions every 2 weeks, in groups of up to 15 professionals, conducted at the participants’ place of work and during their hours of work, by a certified and experimented MM trainer (5 months); daily practice of 10 to 20 minutes was recommended, accompanied with an audio tool adapted to the program. If the number of volunteers exceeded the maximum allowed, measures were taken to ensure equitable representation among the various professional categories.

The MM trainer was external to both the hospital and the initial step of the study. The most appropriate time slots were proposed by the department.

The MM program was derived from the Caring Mindfulness-Based Approach for Seniors (CMBAS) program [55]. The CMBAS follows the general format of an MBSR program, consisting of an initial one-on-one interview; 8 weekly, 2-hour, group-based sessions; and a half day of meditation practice in the sixth week of the program. It is specifically tailored to the needs of older adults, building on modifications suggested by Zellner Keller et al [56] together with a focus on compassion and loving-kindness meditation. Older adults and hospital professionals share a common experience characterized by human and existential vulnerability—encompassing their relationship to finitude and death, physical decline and illness, and the pursuit of meaning amid these experiences [57]. For the PROMIND pilot study, we customized its psychoeducational and practice components, its duration, and number of sessions to be adapted to the population of hospital professionals. Practical exercises were proposed to help them with their emotions, thoughts, and feelings. Participants were asked to engage in home practice, which consisted of formal guided meditations and informal practices aimed at helping to generalize mindfulness skills to daily life. Audio recordings of guided meditation based on the in-person sessions were made available on the hospital’s e-learning support platform (with monitoring of each participant’s connections); additional audio recordings may be proposed according to the needs perceived by participants as the program progresses, selected to match the proposed sessions.

### Evaluation and Data Collection

#### Overview

In a realist randomized trial protocol (Figure 2), the evaluation perspective blends epidemiologic and realist approaches, according to the notion of methodological pluralism [44,49]. A convergent mixed methods experimental design is used with quantitative and qualitative data collected concomitantly and results integrated secondarily at the time of analysis [58]. The complementary paradigms of intervention epidemiology and realist evaluation are thus mobilized in this pilot study to explore and estimate the relevance of the effect measured by the primary exploratory outcome in this specific population and intervention context (recruitment by department/team in a hospital), without confirmatory scope at this stage. Beyond answering the question “Does the intervention work or not?” (limitations of the black box model), realist evaluation enables to answer the question “What works, for whom, how, and under what circumstances?” This information is particularly important for assessing the feasibility of interventions in other contexts and generalizing them beyond pilot departments [59]. In the realist evaluation approach, it is expected to possibly identify CMOCs to explain the effects produced. This is particularly relevant for MBIs in the workplace [42]. In qualitative evaluation, the use of predetermined criteria is inappropriate, as the inductive and exploratory approach seeks to capture the complexity and variability of subjective experiences in various contexts. Thus, qualitative data were collected via the observation guide, the interdepartment focus group (FG) guide, the intradepartment FG guide, and the individual interview guide (Table 1). Quantitative data were collected using self-administered questionnaires (Table 2); adverse events related to the practice, questionnaires, and interviews were collected at the same time on dedicated forms. Quantitative data collection and storage were carried out by a dedicated clinical research associate using a secure electronic case report form (eCRF).

**Table 1.** Schedule for qualitative evaluations in the PROMIND study.

	Study stage						
	Inclusion-baseline	—^a^	Allocation	—			End of the study
Time point	T0^b^	T1^c^	—	T2^d^	—	T3^e^	T4^f^
Months	M1^h^-M6	M6	—	M7 ± 15 d^g^	—	M12 ± 30 d	M15 ± 30 d
Enrollment	✓	—	—	—	—	—	—
Eligibility screen	✓	—	—	—	—	—	—
Informed consent	✓	—	—	—	—	—	—
Allocation	—	—	✓	—	—	—	—
Qualitative assessments							
Interdepartmental FG^i^	—	—	—	✓	—	✓	—
Nonparticipant observation of MM^j^ session	—	—	—	—	✓	—	—
Individual interviews	—	—	—	—	—	✓	—
Intradepartmental FG	—	—	—	—	—	—	✓

a Not applicable.

b T0: the baseline.

c T1: after the MM information meeting/before the MM test session.

d T2: after the MM test session.

e T3: after the end of the MM program.

f T4: 3 months after the end of the MM program.

g d: days.

h M: months.

i FG: focus group.

j MM: mindfulness meditation.

**Table 2.** Schedule for quantitative evaluations in the PROMIND study.

	Study stage					
	Inclusion-baseline	—^a^	Allocation	—		End of the study
Time point	T0^b^	T1^c^	—	T2^d^	T3^e^	T4^f^
Months	M1-M6^g^	M6	—	M7 ± 15 d^h^	M12 ± 30 d	M15 ± 30 d
Enrollment	✓	—	—	—	—	—
Eligibility screen	✓	—	—	—	—	—
Informed consent	✓	—	—	—	—	—
Allocation	—	—	✓	—	—	—
Quantitative assessments						
Sociodemographic characteristics: questions	✓	—	—	—	—	—
Psychological flourishing in the workplace: EEPMT-13^i^	✓	—	—	—	✓	✓
Mindfulness: FFMQ-15^j^	✓	—	—	—	✓	✓
Hostility/aggression/anger: AQ-12^k^	✓	—	—	—	✓	✓
Impulsivity: VAS^l^	✓	—	—	—	✓	✓
Perceived stress: Chamoux-Simard Scale	✓	—	—	—	✓	✓
Patient support: VAS	✓	—	—	—	✓	✓
Credibility and expectations of MM^m^ intervention: credibility and expectations adapted	✓	✓	—	✓^n^	✓^n^	✓^n^
Occupational stress (working conditions-social support at work): Job Content Questionnaire	✓	—	—	—	✓	✓
Experience of conflict-related disturbance: VAS	✓	—	—	—	✓	✓
Evaluation of collective activity: VAS	✓	—	—	—	✓	✓
Evaluation of organizational leeway: VAS	✓	—	—	—	✓	✓
Psychological safety: VAS	✓	—	—	—	✓	✓
MM practice time (outside MM sessions): questions	✓	—	—	✓^n^	✓	✓

a Not applicable.

b T0: the baseline.

c T1: after the MM information meeting/before the MM test session.

d T2: after the MM test session.

e T3: after the end of the MM program.

f T4: 3 months after the end of the MM program.

g M: months.

h d: days.

i EEPMT-13: Echelle Epanouissement Psychologique Milieu de Travail-13.

j FFMQ-15: Five Facets Mindfulness Questionnaire-15.

k AQ-12: Aggression Questionnaire-12.

l VAS: Visual Analog Scale.

m MM: mindfulness meditation.

n Intervention group (IG) only.

#### Data Collection Schedule

The study schedule for the quantitative component (Table 2) consisted of the following steps:

1. An initial step for the 8 departments, comprising the inclusion visit and initial data collection at the baseline (T0) and an information meeting on MM followed by an assessment of MM representations (after the MM information meeting/before the MM test session; T1), followed by the randomization of departments;
2. An MM practice intervention for the 4 departments in the intervention group, comprising an MM test session followed by an evaluation of MM representations (T2) and the MM program; and
3. A follow-up step for the participants of the 8 departments, with measurements of outcomes at the end of the MM program (T3) and at 3 months after the end of the program (T4).

Sociodemographic, professional, psychological, and health data were collected at T0.

The study schedule for the qualitative component in the intervention group (Table 1) comprised the following:

1. FG:

- Interdepartment or longitudinal 1-hour FG with department managers were conducted prior to the MM program (T2) and after the MM program (T3, within 1 month after the end of the MM program) following the individual interviews. During the MM program, an additional FG might be conducted at the request of department managers in order to address any emerging needs related to the implementation. The objectives and themes were to explore and describe the barriers and facilitators to the implementation of the intervention and the group dynamics in each department; explore the variety of contexts in each department; identify the rapid adaptations that would be necessary due to barriers that could compromise implementation; identify the evolution of group dynamics and the mechanisms promoting or compromising implementation; and identify the mechanisms and effects of the intervention and their sustainability.
- Four 1-hour intradepartmental FG were held at T4 with all the volunteer participants. The objectives and themes were to explore and describe the barriers and facilitators to implementation as well as the group dynamics in each department and to explore and describe in depth the mechanisms and effects produced by the intervention. This included assessments at the team level and in the care relationship with patients and/or families.

1. Nonparticipant observation of MM session: during the MM sessions, 1 to 3 observations were conducted per department during the first month and before the end of the MM program. The objectives and themes were to observe the material conditions of the sessions; observe the attitudes and behaviors of professionals during the sessions; observe group dynamics during the sessions with the trainer; and observe nonverbal interactions between participants and with the trainer.
2. Individual interview: a 1-hour semistructured individual interview was proposed after T3, on a voluntary basis, to participants from each department. The objectives and themes were to explore the psychic mechanisms involved in the MM intervention and to explore the effects of the intervention on the relational, managerial, and work organization dimensions. Data saturation was expected to be reached with approximately 15 to 20 participants; the theoretical maximum sample size was 60 participants. Efforts were made to ensure sex, age, and profession balance among participants. Data collection was discontinued once data saturation was deemed sufficient (Table 1).

The topic guides are provided in Multimedia Appendix 1.

#### Study Outcome Measures

The primary outcome measure was the evolution of psychological flourishing at work (EEPMT-13) at T0 and at T3. It was measured using the adapted and validated French-language scale Psychological Flourishing at Work (EEPMT-8) [9], to which the Professional Life Satisfaction Scale (ÉSVP-5) [60] was added to form a new 13-item Psychological Flourishing at Work Scale (EEPMT-13).

The secondary outcome measures were as follows:

- Evaluation of the impact of the MM program between T0, T3, and T4 at (1) the individual level and (2) the department level: at the individual level, mindfulness was measured using the Five Facets Mindfulness Questionnaire (FFMQ-15; 15 items) [61], hostility, aggression, or anger was measured by the Aggression Questionnaire (AQ-12; 12 items) [62]; impulsivity was measured using a Visual Analog Scale (VAS); perceived stress was measured using the Chamoux-Simard Scale (3 questions as a VAS) [63]; the experience of being disturbed by conflict was measured using a VAS; and patient support was measured using a VAS. At the department level (collective level), job strain was measured using the Job Content Questionnaire (26 items) [64]; collective activity was measured using Likert scales (4 items) [65-67]; organizational leeway was measured using Likert scales (4 items) [68]; and psychological safety at work was measured using a VAS.

- The evolution of professionals’ representation of MM:

1. For all participants, it was measured between T0 and T1 using the credibility and expectancy questionnaire (adapted Devilly and Borkovec Credibility/Expectancy Questionnaire; 6 items) [69].
2. For participants in the intervention group, the evolution of MM representation between T1, T2, T3, and T4 was measured using the credibility and expectancy questionnaire (adapted Devilly and Borkovec Credibility/Expectancy Questionnaire; 6 items) [69].

- Description of professionals’ representations of MM and their evolution: the description was obtained through observation reports (during the MM program) and through verbatim responses from interdepartmental FG before the program, from semistructured interviews (T3, after quantitative evaluation), from interdepartmental FG after the MM program (T3, after quantitative evaluation), and from intradepartmental FG away from the MM program (T4, after quantitative evaluation).
- Description of the implementation of the MM intervention: the implementation was described in terms of fidelity (number of sessions conducted, number of participants in sessions, duration of sessions, and daily practices); barriers and facilitators to the implementation of the MM program in the context of hospital departments, identified using verbatim responses from the FG and semistructured interviews; and observation reports as well as adherence to the intervention in terms of attendance at in-person practice sessions, time spent practicing MM outside sessions, and daily life practice (3 items: number of formal and informal sessions per week and time spent at T0, T3, and T4; monitoring by e-learning support).
- Exploration and description of the effects of the intervention and the mechanisms by which they are produced in different departments, looking for CMOCs
- Compliance with the intervention: deviations from the protocol were tracked and reported in terms of adherence to the protocol (sessions taking place, schedule respected), by tracking attendance at program sessions, accessing audio support material between sessions, and the MM practice outside sessions. The MM trainer is attentive to group dynamics and provides feedback to the study team.

The English translation of the PROMIND questionnaire (original in French) is provided in Multimedia Appendix 2.

### Data Analysis

#### Quantitative Analysis

##### Number of Subjects

As this is a pilot study, the number of participating departments (N=8) and the number of professionals studied (15 professionals per department; N=120) were not determined by a power calculation [70] but by the operational constraints of the study, notably the maximum number of subjects recommended by the MM trainer for a group of meditative practice [55,71,72].

##### Statistical Methods

The primary objective of this study is to compare the evolution (between T0 and T3) in psychological flourishing at work in the intervention group compared with the control group.

###### Analysis of the Primary Outcome

For each individual, the psychological flourishing at work was measured (using EEPMT-13) at 2 time points: T0 and T3. Since each individual has 2 measures, and each individual belongs to a given department, we will use a multilevel model for the analysis of the primary outcome [73]. These models, also known as mixed models, are particularly well-suited to the analysis of this type of hierarchical data (ie, first level=the measure, second level=the individual, and third level=the department).

Noting Y_ijk_ as the EEPMT-13 measure j of individual i in department k, we obtain the following model 1 equation:


$$Y_{ijk}=\beta_{0}Z_{k}+u_{ik}+v_{k}+\beta_{1}{\;}^{*}X_{ijk}+\beta_{2}X_{ijk}G_{k}+e_{ijk}$$

where u_ik_, v_k_, and e_ijk_ are, respectively, the individual random effect (individuals having greater or lesser flourishing), the department random effect (certain departments presenting on average greater or lesser levels of flourishing), and the residual deviation of the ijk measure; Z_k_ is a vector of binary indicator variables indicating the department type of department k; G_k_ is an indicator variable for the intervention group (0 if the department belongs to the control group and 1 if the department has received the intervention); X_ijk_ is a measurement time indicator variable (0 if the measurement is taken at T0 and 1 if it is carried out at T3).

With this coding, the 4 fixed parameters making up the β_0_ vector provide mean levels according to the department type (which is a stratification factor in randomization), the parameter β_1_ corresponds to the variation between T0 and T3 for departments belonging to the control group, and β_1_ + β_2_ corresponds to the variation between T0 and T3 for departments belonging to the intervention group. The parameter β_2_ thus corresponds to the parameter of interest that describes the effect of the intervention, and the main hypotheses tested will be as follows:

H0: evolution between T0 and T3 is identical between the 2 groups (ie, β_2_=0)

H1: evolution between T0 and T3 is not identical between the 2 groups (ie, β_2_≠0)

This test will be performed using the Wald test (2-tailed test, type I error rate=5%).

It will be investigated whether a residual effect of the intervention persists at T4, by analyzing the 2 measurements at T0 and T4 in a model similar to model 1.

###### Analysis of Secondary Outcomes

Quantitative secondary outcomes (overall score and questionnaire dimensions) will be described for the population, by demographic variable modality and by intervention group, using standard descriptive statistics. These quantitative criteria will be compared between the 2 groups using the Student *t* test or the Wilcoxon-Mann-Whitney test, according to the distribution.

Categorical secondary outcomes will be summarized for the total population, by demographic variable modality and by intervention group, using the following descriptive statistics: frequencies and percentages for each variable level and for missing values. These categorical criteria will be compared between the groups using the Chi-square test or Fisher exact test, according to the distribution.

###### Missing Data and Software

Every effort was made to minimize the number of missing data. If there are still missing data points, these will be replaced at T0 by the mean of the available data in each group (intervention or control group).

Statistical analysis will be carried out using R software (version 4.5.3; R Foundation for Statistical Computing)) [74].

### Qualitative Data Analysis

#### Data Management and Analysis

Pseudonymized transcripts will be produced. Qualitative data will be systematically coded. Triangulation of coding will be conducted for the initial FG and interviews to ensure consistency in coding.

#### Deductive and Inductive Analysis

Data from FG and individual interviews will undergo thematic content analysis (MAXQDA 2020 software [75]). Analyses will follow a deductive approach (based on the themes from the data collection guides), followed by an inductive approach (developing new categories to account for all collected data), with iteration. The analysis will consider 4 levels: individual, team or department, organization or hospital, and the external hospital context (health care system). Data collected through observations will be analyzed and used to contextualize the analysis of FG and interview data and to triangulate the findings. Qualitative data will be analyzed using a combined deductive-inductive approach within a realist evaluation framework. A theoretical framework of intervention implementation will inform the initial coding structure, which will be subsequently refined inductively to incorporate data generated beyond predefined categories. Reflexivity will be ensured through field notes and iterative multidisciplinary team discussions, enhancing credibility and confirmability. Reliability will be reinforced using qualitative data analysis software, ensuring traceability of the analytic process and supporting confirmability [76].

### Integration of Findings

Qualitative and quantitative data will be analyzed separately. Integration will be performed subsequently to identify points of convergence and divergence and to generate hypotheses for further analysis. We will apply a table of convergences and divergences between the results, the interpretation of which will be discussed by the interdisciplinary team and compared to the literature. Feasibility as an outcome will be evaluated according to an overall informed decision-making framework [44], based on prespecified indicators and conclusions based on a realist approach: exploratory outcomes of the intervention, feasibility, and barriers and facilitators identified for each context; consistency with the theoretical logic model; and potential for organizational adaptation. They will be analyzed in each department context to inform trends and assess the barriers and facilitators to inform future scalability. This integrated analysis, rather than isolating the components, will provide a more comprehensive assessment of effectiveness, feasibility, and implementation.

### Ethical Considerations

This study was approved by the Sud-Est VI ethics committee (Clermont Ferrand, France) and the French national data protection authority (*Commission nationale de l’informatique et des libertés*, CNIL). The study was registered on ClinicalTrials.gov (NCT06331065). Written informed consent was obtained from participants, as required by French law for this type of study. All participants were informed of the study objectives and data confidentiality. Information was provided to the HCL Social and Economic Committee training group.

This protocol has been described in compliance with the SPIRIT (Standard Protocol Items: Recommendations for Interventional Trials) 2025 checklist (Checklist 1) [77].

## Results

The PROMIND study was funded in October 2021, and the funding was extended in June 2024. This study was conducted from May 2024 to June 2025. In total, 108 participants were enrolled in the study; data collection ended on June 30, 2025. Data analysis is in progress. After data analysis, the COPR will be asked to discuss the results and prospects for implementing MM based on the study findings. Workers’ representatives will be informed of the results. A dedicated meeting to summarize and discuss the results of PROMIND will be planned with the participants.

We expect the results of this study to be published by the end of 2026. Scientific publications and communications (national and international) will be planned to publicize this trial and its findings in the fields of psychiatry, public health, and occupational health. Recommendations for publication in the field of epidemiology and realist evaluation will be followed.

## Discussion

### Principal Findings

This study expects that the co-constructed workplace-based mindfulness intervention is an acceptable and relevant intervention for French hospital professionals: we hypothesize that the implementation of this MBI improves psychological flourishing in the workplace in 4 hospital departments in comparison with a geographical control and, more broadly, mental health at work at the individual and collective levels. With the intervention modalities chosen in accordance with the intervention mapping protocol [53], we expect that this improves the relevance of the study and the feasibility of its implementation.

The originality of the PROMIND study is the adoption of a structured approach to intervention planning in mental health promotion for hospital professionals. The strengths of this innovative study lie in the improvement strategies employed, including the co-construction, and the collective approach to the intervention and to the MM program within a team that usually works together (department) [78]. Moreover, this MBI is a multiprofessional training course that also transmits practical resources and is prepared as an organizational intervention, taking into consideration the views of professionals, which is conducive to team effectiveness [79]. The participatory development with stakeholders is one of the recommendations for promoting the feasibility of implementation, the relevance, and ultimately the sustainability of the proposed intervention [44,53]. The initial co-construction step enabled the emergence of local “ambassadors” within the participating departments involved in relaying study information and their motivation; the identification of main MBI components, such as professional time that could be allocated to training (eg, interval and duration of sessions); and the communication strategies to support participation. This also facilitated the support and practical involvement of hospital executive management throughout the study, and it is known that culture and infrastructure also improve feasibility [45]. In addition, a reduced duration of practice sessions was chosen for this MBI adapted for hospital professionals. While brief interventions can facilitate access to a meditation training program, the dose proposed must be sufficient to ensure effectiveness [41] and requires specific evaluation.

The innovative feature of this study is the adoption of a mixed evaluation methodology. The methodological pluralism is based on a randomized controlled pilot study with a complementary approach of realist evaluation, due to the complex nature of the intervention [44]. Intervention epidemiology raises the challenge of choosing a primary outcome, whereas mental health is a complex domain, which requires the use of several questionnaires for a reliable assessment. The EEPMT-13 is an innovative tool to specifically explore mental health in the workplace, which requires further exploration. The focus of the evaluation on positive mental health opens up a viewpoint that complements the approaches to distress at work, to adopt a broader view of mental health as a continuum [80,81]. Most studies investigating MM among hospital professionals investigate burnout using questionnaires such as the Maslach Burnout Inventory Scale as a specific tool for assessing distress in the workplace [41]. However, some consider that it is not based on a solid theoretical model and that the construction of certain items is questionable [82]. Otherwise, they use non–workplace-specific and routine mental health assessment criteria (eg, anxiety, stress, and quality of life) [41]. The complementary realist evaluation approach provides a deeper and sharp understanding of this complex domain at the individual and organization levels and in the patient care relationship [59]. Although this pilot study may not provide conclusive evidence, it will provide useful knowledge on how these interventions work in a real-world hospital setting and on the extent to which MM can promote mental health in an organizational environment with psychosocial risk factors. Finally, this mixed methods design is especially appropriate for pilot studies of complex interventions where the exploratory primary outcome of effectiveness as well as feasibility and processes must be explored.

### Limitations

In this pilot study, inclusion was limited to hospital care departments. However, hospitals include technical and administrative departments with specific needs that may require adaptation of the intervention. This will require a new dedicated study. In addition, while all willing professionals from participating departments can be included, only part of the team can participate in the MM program because the number of people is limited per group (n=15). Moderately sized groups are therefore usual in MBI, and group size varies according to the program and practice contexts [55,71,72]. This limits the potential impact of MBI on the departments and work organization, which will be assessed through realist evaluation. In a larger-scale study, it may be interesting to train more professionals at the same time in each department, based on the evaluations of this pilot study. It would also be important to include more departments in a larger-scale study. Indeed, in this pilot study, only 2 groups comprising 4 departments each could be included. Any associations identified in this study should therefore be interpreted with caution due to the risk of bias and will need to be confirmed during the scalability stage. Furthermore, the demands associated with the mixed methods evaluation in the study, particularly in terms of the time required, complicate the implementation of an intervention among a specific population for whom the issue of availability and task prioritization is a major concern. The self-administered questionnaires may induce a desirability bias [83]. However, the quantitative assessment is fast. In addition, the self-administered questionnaires can reduce anxiety during the assessment and investigate behaviors that are difficult to observe in other ways [84]. This pilot study has an open-label design, which may introduce bias. The delivery of the MM sessions by an external trainer limits the impact of the lack of blinding during the MM program.

### Conclusion

The results of this pilot study will provide essential data and input for the sustainable and larger-scale implementation of MBIs in the workplace, facilitating access to relevant, evidence-based interventions to support the mental health of hospital professionals. The challenges of implementation and evaluation of a workplace-based mindfulness intervention adapted to hospital professionals are multiple and complex; the use of a health program planning model such as the intervention mapping protocol is important for structuring the steps of co-construction, implementation, and evaluation of the intervention. Participative research and cooperation between hospital professionals, researchers, experts in MM, and the hospital management are essential to promote the success of initiatives such as this.

## Supplementary material

10.2196/80725 Multimedia Appendix 1 Topic guides.

10.2196/80725 Multimedia Appendix 2 English translation of the PROMIND Questionnaire.

10.2196/80725 Checklist 1 SPIRIT (Standard Protocol Items: Recommendations for Interventional Trials) 2025 checklist.

## Abbreviations

AQ-12: 12-item Aggression Questionnaire
CMBAS: Caring Mindfulness-Based Approach for Seniors
CMOC: context-mechanism-outcome configuration
CNIL: French national data protection authority (Commission nationale de l'informatique et des libertés)
COPR: Committee of Participants’ Representatives
eCRF: electronic case report form
EEPMT-13: 13-item Psychological Flourishing at Work Scale
EEPMT-8: Psychological Flourishing at Work Scale
ÉSVP-5: Professional Life Satisfaction Scale
FG: focus group
HCL: Hospices Civils of Lyon
MBCT: mindfulness-based cognitive therapy
MBI: mindfulness-based intervention
MBSR: mindfulness-based stress reduction
MM: mindfulness meditation
MRC: Medical Research Council
SPIRIT: Standard Protocol Items: Recommendations for Interventional Trials
VAS: Visual Analog Scale
