# Supplementary material for: Promoting the Mental Health of Hospital Professionals Through a Workplace-Based Mindfulness Intervention: Protocol for a Randomized Controlled Pilot Trial With a Realist Evaluation (PROMIND)
Source: JMIR Res Protoc. 2026 Aug 18;15:e80725. doi: 10.2196/80725 (PMC13487142; doi:10.2196/80725)
Supplement: Supplementary file 1 [file resprot-v15-e80725-s002.pdf]

**PROMIND Questionnaires (administered in French - translation for the publication)**

**SOCIO-DEMOGRAPHIC CHARACTERISTICS**

Sex: ☐ Male ☐ Female ☐ Do not wish to answer

How old are you (years)?

☐ Under 20 ☐ Between 20 and 29 ☐ Between 30 and 39

☐ Between 40 and 49 ☐ Between 50 and 59 ☐ Over 60

Do you live with a partner (married or cohabiting)? ☐ Yes ☐ No

How many children do you have? |__|__|

What's your profession?

☐ Non-medical staff nurses ☐ Junior medical staff ☐ Administrative staff

☐ Non-medical staff nursing assistants ☐ Senior medical staff ☐ Technical staff

☐ Non-medical staff, neither nurses nor nursing assistants ☐ Other: ……………………………………………….

Are you in a supervisory/managerial position? ☐ Yes ☐ No ☐ Do not wish to answer

How long have you been with HCL (Hospices Civils of Lyon) (years)?

☐ Less than a year ☐ Between 1 and < 3 years ☐ Between 3 and < 5 years ☐ Between 5 and < 10 years ☐ 10 years or more

Do you have any previous experience of mindfulness meditation?

☐ None / one-off ☐ Moderate* practice ☐ Sustained** practice

Do you have any previous experience with yoga/qi gong/tai chi?

☐ None / one-off ☐ Moderate* practice ☐ Sustained** practice

Free commentary (tradition/formation of practice(s), seniority, average daily duration, etc.): ………………………………………………………………………………………………………………..

*practice at least several times a week for at least 10 minutes/day, for at least 3 months

**daily practice of at least 40 minutes/day for at least 3 months and at least one intensive retreat/training (i.e. more than 5 hours of daily practice)

Do you have a current somatic health problem? ☐ Yes ☐ No ☐ Do not wish to answer

Do you have a current mental health or addiction problem? ☐ Yes ☐ No ☐ Do not wish to answer

Are you currently taking any medication? ☐ None ☐ Psychotropic ☐ Other medication ☐ Do not wish to answer

How do you feel about your health at the moment? (place a cursor or cross on the line) ☐ Do not wish to answer

Bad |———————————————| Good

Are you currently experiencing any psychosomatic difficulties (pain, fatigue, functional bowel problems, etc.)? (place a cursor or cross on the line) ☐ Do not wish to answer

Not at all |———————————————| Completely

**PSYCHOLOGICAL FLOURISHING AT WORK**

This questionnaire concerns your professional life. Below are several statements with which you may more or less agree (or disagree). Indicate your position (agree or disagree) by circling the answer that best expresses your point of view. Be spontaneous and sincere in your answers.

| | **Strong disagree** | **Disagree** | **Rather disagree** | **Neither agree or disagree** | **Rather agree** | **Agree** | **Strongly agree** |
|---|---|---|---|---|---|---|---|
| **1.** In general, my professional life is very much in line with my ideals | 1 | 2 | 3 | 4 | 5 | 6 | 7 |
| **2.** My working conditions are excellent | 1 | 2 | 3 | 4 | 5 | 6 | 7 |
| **3.** I am satisfied with my professional life | 1 | 2 | 3 | 4 | 5 | 6 | 7 |
| **4.** So far, I've achieved the important things I wanted in my professional life | 1 | 2 | 3 | 4 | 5 | 6 | 7 |
| **5.** If I could start my professional life all over again, I would change almost nothing | 1 | 2 | 3 | 4 | 5 | 6 | 7 |
| **6.** I lead a professional life that has purpose and meaning | 1 | 2 | 3 | 4 | 5 | 6 | 7 |
| **7.** My social relationships at work are supportive and rewarding | 1 | 2 | 3 | 4 | 5 | 6 | 7 |
| **8.** I'm involved and interested in my daily and professional activities | 1 | 2 | 3 | 4 | 5 | 6 | 7 |
| **9.** I actively contribute to the happiness and well-being of others | 1 | 2 | 3 | 4 | 5 | 6 | 7 |
| **10.** I am competent and diligent in the professional activities that are important to me | 1 | 2 | 3 | 4 | 5 | 6 | 7 |

| | | | | | | | |
|---|---|---|---|---|---|---|---|
| **11.** I am a “good” person with a “good” professional life | 1 | 2 | 3 | 4 | 5 | 6 | 7 |
| **12.** I am optimistic about my professional future | 1 | 2 | 3 | 4 | 5 | 6 | 7 |
| **13.** People respect me in my work | 1 | 2 | 3 | 4 | 5 | 6 | 7 |

**FFMQ-15**

Gu J, Strauss C, Crane C, Barnhofer T, Karl A, Cavanagh K, et al. Examining the factor structure of the 39-item and 15-item versions of the Five Facet Mindfulness Questionnaire before and after mindfulness-based cognitive therapy for people with recurrent depression. Psychol Assess. 2016 July;28(7):791–802.

**PERCEIVED STRESS (Chamoux and Simard scale)**

Lesage FX, Berjot S. Validity of occupational stress assessment using a visual analogue scale. Occup Med (Lond). 2011 Sept; 61(6):434–6.

**JOB CONTENT QUESTIONNAIRE**

Niedhammer I, Ganem V, Gendrey L, David S, Degioanni S. Propriétés psychométriques de la version française des échelles de la demande psychologique, de la latitude décisionnelle et du soutien social du « Job Content Questionnaire » de Karasek : résultats de l’enquête nationale SUMER. 2006;18:413–27.

**CONFLICT DISTURBANCE**

At what level do you currently feel overwhelmed during a conflict at work? (Place a cursor or cross on the line)

Not at all |————————————————| Completely

**ASSESSMENT OF COLLECTIVE ACTIVITY AND ORGANIZATIONAL LEEWAY**

Within our team, we enjoy a certain degree of autonomy, in agreement with the other members of the team.

| 0 | 1 | 2 | 3 | 4 | 5 | 6 | 7 | 8 | 9 | 10 |
|---|---|---|---|---|---|---|---|---|---|---|
| O | O | O | O | O | O | O | O | O | O | O |
| *Strongly disagree* | | | | | | | | | | *Strongly agree* |

I trust my colleagues in the same profession

| 0 | 1 | 2 | 3 | 4 | 5 | 6 | 7 | 8 | 9 | 10 |
|---|---|---|---|---|---|---|---|---|---|---|
| O | O | O | O | O | O | O | O | O | O | O |
| *Strongly disagree* | | | | | | | | | | *Strongly agree* |

I trust my colleagues in different professions

0 1 2 3 4 5 6 7 8 9 10
Strongly disagree — Strongly agree

I sometimes take care of a colleague who's experiencing disturbances

0 1 2 3 4 5 6 7 8 9 10
Strongly disagree — Strongly agree

You are involved in improving work organization or work processes in your department or organization

☐ Always ☐ Most of the time ☐ Often ☐ Rarely ☐ Never

I can't do a good job, I have to sacrifice quality

☐ Never ☐ Sometimes ☐ Often ☐ Always

I have to hide my emotions or pretend to be in a good mood

0 1 2 3 4 5
Strongly disagree — Strongly agree

Do you have the opportunity to discuss organizational or operational issues with others in your workshop or department?

0 1 2 3 4 5
Strongly disagree — Strongly agree

**PSYCHOLOGICAL SAFETY AT WORK**

Psychological safety is the workplace climate in which professionals can express themselves, in which they feel at ease. They can freely share their ideas, even embryonic ones, their concerns, their questions and their mistakes, without fear of being judged or retaliated against.

What is your current level of psychological safety at work? (place a cursor or cross on the line)

Not at all |———————————————| Completely

**PATIENT SUPPORT**

What level of support do you give patients? (place a cursor or cross on the line)

Not at all |———————————————| Completely

**AQ-12**

Here's a series of 12 statements. For each one, indicate whether it characterizes you (“Definitely me”) or not (“Not me at all”). Answer each question as honestly as possible.

| | | |
|---|---|---|
| 1. | Given enough provocation, I may hit another person. | Not at all like me ☐ 1 ☐ 2 ☐ 3 ☐ 4 ☐ 5 ☐ 6 Completely like me |
| 2. | I often find myself disagreeing with people. | Not at all like me ☐ 1 ☐ 2 ☐ 3 ☐ 4 ☐ 5 ☐ 6 Completely like me |
| 3. | I flare up quickly but get over it quickly. | Not at all like me ☐ 1 ☐ 2 ☐ 3 ☐ 4 ☐ 5 ☐ 6 Completely like me |
| 4. | At times I feel I have gotten a raw deal out of life. | Not at all like me ☐ 1 ☐ 2 ☐ 3 ☐ 4 ☐ 5 ☐ 6 Completely like me |
| 5. | There are people who pushed me so far that we came to blows. | Not at all like me ☐ 1 ☐ 2 ☐ 3 ☐ 4 ☐ 5 ☐ 6 Completely like me |
| 6. | can't help getting into arguments when people disagree with me. | Not at all like me ☐ 1 ☐ 2 ☐ 3 ☐ 4 ☐ 5 ☐ 6 Completely like me |
| 7. | Sometimes, I fly off the handle for no good reason. | Not at all like me ☐ 1 ☐ 2 ☐ 3 ☐ 4 ☐ 5 ☐ 6 Completely like me |
| 8. | I wonder why sometimes I feel so bitter about things. | Not at all like me ☐ 1 ☐ 2 ☐ 3 ☐ 4 ☐ 5 ☐ 6 Completely like me |
| 9. | I have threatened people I know. | Not at all like me ☐ 1 ☐ 2 ☐ 3 ☐ 4 ☐ 5 ☐ 6 Completely like me |
| 10. | My friends say that I'm somewhat argumentative. | Not at all like me ☐ 1 ☐ 2 ☐ 3 ☐ 4 ☐ 5 ☐ 6 Completely like me |
| 11. | I have trouble controlling my temper. | Not at all like me ☐ 1 ☐ 2 ☐ 3 ☐ 4 ☐ 5 ☐ 6 Completely like me |
| 12. | Other people always seem to get the breaks. | Not at all like me ☐ 1 ☐ 2 ☐ 3 ☐ 4 ☐ 5 ☐ 6 Completely like me |

**IMPULSIVITY**

How impulsive do you feel right now? (place a cursor or cross on the line)

Not at all |———————————————| Completely

**CREDIBILITY AND EXPECTATIONS ADAPTED**

Concerning the proposed intervention (mindfulness meditation):

**Set I**

1. At this point, does this intervention seem logical to you?

1 2 3 4 5 6 7 8 9

Not at all logical somewhat logical very logical

2. At this point, how useful do you think this intervention will be for you to be flourishing at work?

1 2 3 4 5 6 7 8 9

Not at all useful somewhat useful very useful

3. How confident would you be in recommending this program to a colleague?

1 2 3 4 5 6 7 8 9

Not at all confident somewhat confident very confident

4. How much do you think the positive impact on your well-being will be after the intervention?

0% 10% 20% 30% 40% 50% 60% 70% 80% 90% 100%

**Set II**

For this set, close your eyes for a few moments, and try to identify what you really *feel* about the intervention and its likely success. Then answer the following questions:

1. At this point, how useful do you feel this intervention will be for you to work in good conditions?

1 2 3 4 5 6 7 8 9

Not at all useful somewhat useful very useful

2. How much do you think the positive impact on your well-being will be after the intervention?

0% 10% 20% 30% 40% 50% 60% 70% 80% 90% 100%

**TIME SPENT PRACTICING MM OUTSIDE SESSIONS**

How much practice/exercise in MM (*mindfulness meditation*) have you had outside group sessions in the last two weeks?

- average number of MM sessions per week: |__|__|
- average time spent per MM session:

☐ 5 min ☐ 10 min ☐ 15 min ☐ 20 min ☐ more than 20 min

- average number of opportunities for informal practice* per week: |__|__|
  - if done: situations giving rise to informal practice (free response):

……………………………………………………………………………………………………………………

*informal practice: meditative practice (becoming aware of the present moment) at any time of day, spontaneously (e.g. by concentrating on small everyday gestures (eating, walking, opening a door, etc.).
