# Supplementary material for: Promoting the Mental Health of Hospital Professionals Through a Workplace-Based Mindfulness Intervention: Protocol for a Randomized Controlled Pilot Trial With a Realist Evaluation (PROMIND)
Source: JMIR Res Protoc. 2026 Aug 18;15:e80725. doi: 10.2196/80725 (PMC13487142; doi:10.2196/80725)
Supplement: Supplementary file 3 [file resprot-v15-e80725-s001.docx]

**PROMIND topic guides (in French - translation for the publication)**

**Inter-departmental focus group topic guide**

| **Objectives** | **Themes explored** |
| --- | --- |
| Explore and describe the barriers and facilitators to implement the intervention |  |
| At the department level | - History of the department - Physical environment (room, chairs, quiet or not, etc.) - Geographic accessibility (proximity, availability of facilities) - Availability/absenteeism of each category of professional - Perceived or expressed needs at the collective level - Perceived or expressed benefits at the collective level - Perceived or expressed drawbacks at the collective level - Degree to which the MM intervention aligns with the department’s culture - Degree of competition between the MM intervention and other similar interventions - Degree of competition between the MM intervention and other priorities within the department - Intercurrent events affecting program implementation - Other barriers or facilitators at the departmental level |
| At the individual level | - Perceived or expressed needs at the individual level - Perceived or expressed MPC representations at the individual level - Perceived or expressed benefits at the individual level - Perceived or expressed inconveniences at the individual level - Perceived or expressed disagreements at the individual level - Other perceived or expressed barriers or facilitators at the individual level |
| At the hospital level | - Degree to which the MM intervention aligns with the culture of the hospital network, the site, or the HOSPICES CIVILS OF LYON - Degree of competition between the MM intervention and other similar interventions - Degree of competition between the MM intervention and other priorities of the hospital network, the site, or the HOSPICES CIVILS OF LYON - Intercurrent events affecting the program’s implementation |
| Explore and describe the dynamics of the participant group in each department  • During training sessions  • At the end of each training session  • Between training sessions | - Instructor’s impressions - Management’s impressions - Leadership (individual) - Collective leadership - Implementation of mindfulness practice time between training sessions (in the department or elsewhere) - Respect for the confidentiality of exchanges - Freedom of exchange (challenges of hierarchical vs flat management attitudes) |
| Explore and describe the potential future of MM practice in each department | - Barriers or facilitators for continuing the practice after the intervention |

**Intra-departmental Focus Group topic guide**

| **Objectives** | **Themes explored** |
| --- | --- |
| Explore and describe the barriers and facilitators to implement the intervention |  |
| At the department level | - History of the department - Physical environment (room, chairs, quiet or not, etc.) - Geographic accessibility (proximity, availability of facilities) - Availability/absenteeism of each category of professional - Perceived or expressed needs at the collective level - Perceived or expressed benefits at the collective level - Perceived or expressed drawbacks at the collective level - Degree to which the MM intervention aligns with the department’s culture - Degree of competition between the MM intervention and other similar interventions - Degree of competition between the MM intervention and other priorities within the department - Intercurrent events affecting program implementation - Other barriers or facilitators at the departmental level |
| At the individual level | - Perceived or expressed needs at the individual level - Perceived or expressed benefits at the individual level - Perceived or expressed inconveniences at the individual level - Perceived or expressed disagreements at the individual level - Other perceived or expressed barriers or facilitators at the individual level |
| At the hospital level | - Degree to which the MM intervention aligns with the culture of the hospital network, the site, or the HOSPICES CIVILS OF LYON - Degree of competition between the MM intervention and other similar interventions - Degree of competition between the MM intervention and other priorities of the hospital network, the site, or the HOSPICES CIVILS OF LYON - Intercurrent events affecting the program’s implementation |
| Explore and describe the dynamics of the participant group in each department  • During training sessions  • At the end of each training session  • Between training sessions | - Impressions of the participants - Leadership (individual) - Collective leadership - Implementation of mindfulness practice time between training sessions (in the department or elsewhere) - Respect for the confidentiality of exchanges - Freedom of exchange (challenges of hierarchical vs flat management attitudes) |
| Explore and describe the potential future of MM practice in each department | - Barriers or facilitators for continuing the practice after the intervention |
| Explore and describe in depth the mechanisms and outcomes of the intervention:  (a) at the team level (work relationships and organization, quality of collaboration, etc.)  (b) in the healthcare relationship with patients and/or families | (a) At the team level:   - Presence for others (colleagues) - Kindness and compassion to others (in daily situations, or during challenging, stressful, or conflicting situations) - Inter-professional collaboration - Mutual support in tasks or work activities (in daily situations or stressful situations) - Emotional regulation in interpersonal relationships; managing situations involving aggression, tension, and conflict - Social sharing of positive emotions - Sense of belonging to a teamwork - Sense of coherence, satisfaction, and flourishing in the work done - Quality and safety of healthcare - Changes in work organization, task management, and task execution   (b) In the healthcare relationship with patients and/or families   - Ability to be present and tuned to the patient in the relationship - Ability to understand the patient (empathy) - Emotional regulation - Experiences of perceived stress, compassion fatigue, and well-being at work - Development of healthcare provider skills: warmth, commitment, authenticity, attentiveness |

**Individual Interview topic guide**

| **Objectives** | **Exploratory themes** |
| --- | --- |
| Explore the characteristics of personal MM practice, including barriers and facilitators | - Formal practice: alone / with others, frequency, duration, location, material(s) - Informal practice: circumstances/locations, frequency, duration - Barriers - Facilitators |
| Explore the mental and individual mechanisms involved in the mindfulness meditation intervention | - Attention regulation: ability to be mindful, to maintain or bring attention back to the present moment - Decentration: cognitive defusion, shift in perspective, decreased rumination - Emotional regulation: getting to know one’s emotions, acceptance, non-judgment, kindness - Body awareness - Changes in well-being, and possible burnout, compassion fatigue, or perceived stress - Possible issues resurfacing, and managing past personal situations - Representations of MM |
| Explore the effects of the intervention on interpersonal relationships:   - With those close to them - With work colleagues/management - With patients - With people in general | - Ability to be present with someone else - Ability to understand someone else (cognitive empathy, emotional empathy) - Ability to attune to someone else in the relationship, emotional regulation, behaviors within the relationship - Development of human skills: warmth, commitment, authenticity, mindfulness |
| Explore the effects of the intervention on the managerial level   - From the perspective of management - From the perspective of team members | - Management practices: autonomy, communication, delegation, collaboration (decision-making mechanisms) - Opportunities (times/locations) for sharing about work, the meaning of work, and teamwork building - Aspiration to lead and to serve |
| Explore the effects of the intervention on the organization and realization of work | - Inter-professional collaboration, cooperation - Mutual support in work tasks or situations (in routine or stressful situations) - Regulation of emotions in relationships, management of aggression, tensions, and conflicts - Social sharing of positive emotions - Sense of belonging to a teamwork - Sense of coherence, satisfaction, and fulfillment in the work done - Quality and safety of healthcare - Changes in work organization, task assignment, and task realization |

**Non-participant observation de MM session**

| Observe the material conditions of meditation sessions | 1. room  2. number of chairs, chair arrangement, type of chairs  3. atmosphere: quiet or not, lighting, etc.  4. instructor’s seating arrangement, participants’ seating arrangement (mix of professions or not, gender), gender diversity |
| --- | --- |
| Observe the (non-verbal) attitudes and behaviors of professionals during sessions | 1. relaxed: slower breathing, jokes ...  2. tense: heavy silence, stiffness ...  3. behavioral anxiety (shorter breathing, restlessness...)  4. respect (chatter etc.), indifference  5. postures on the chair, facial features, reactions before/during/after the practice (sighing, tears etc.)  6. posture of the hierarchy: present, erased... |
| Observe group dynamics during sessions with the instructor | 1. presence of questions  2. followed instructions  3. shares: feedback, question, self-sharing  4. relationship to management, perceived team cohesion (imitative behavior of a colleague, mutual aid, seeking/providing spontaneous support) |
| Observe the non-verbal interactions between participants and with the instructor | 1. gaze of the participants  2. participants' behavioral responsiveness (smiling, etc.)  3. group alliance and instructor |

MM: Mindfulness Meditation
